# Does disease management for diabetic nephropathy reduce medical expenditure? Evidence from a three-period difference-in-differences analysis

**DOI:** 10.1186/s12913-020-05297-0

**Published:** 2020-05-11

**Authors:** Hiroyuki Kawaguchi, Michiko Moriyama, Hideki Hashimoto

**Affiliations:** 1 Seijo University, Faculty of Economics, 6-1-20 Seijo, Setagaya-ku, Tokyo, 157-8511 Japan; 2 Clinical Nursing Research Laboratory, Chronic Care and Family Nursing Unit, Hiroshima University, Graduate School of Biomedical & Health Sciences, 1-2-3 Kasumi, Minami-ku, Hiroshima, 734-8553 Japan; 3 Department of Health and Social Behavior, The University of Tokyo, School of Public Health, 7-3-1 Hongo Bunkyo-ku, Tokyo, 133-0033 Japan

**Keywords:** Disease management, Diabetes, Dialysis, Difference-in-differences analysis, Charlson comorbidity index

## Abstract

**Background:**

To discriminatively evaluate the cost-saving effects of a disease management program for diabetic nephropathy patients through care process rectification and, subsequently, improved health outcomes.

**Methods:**

This study links public medical insurance claims data to the health records of a disease management program for diabetic nephropathy patients. To account for selection bias caused by the non-randomized allocation of the disease management program for diabetes patients, we adopted a fixed-effect model of panel data analysis. To discriminatively evaluate the cost-saving effects of the disease management program for diabetic nephropathy patients through care process rectification and, subsequently, improved health outcomes, we expanded the difference-in-differences analysis from the traditional two-period model to a three-period model, comprising the before-intervention, during-intervention, and after-intervention periods. Data were extracted from municipal public insurers in Kure, Japan.

**Results:**

The cost-reduction effect in terms of treatment costs from the before-intervention period to the during-intervention period (the rectification effect) was 4.02%, and the cost-saving effect from the during-intervention period to the after-intervention period (the health improvement effect) was 2.95%.

**Conclusions:**

A disease management program for diabetes patients organized by local public insurers in Japan reduced costs both by amending treatment processes and by subsequently improving the prognosis of the disease.

## Background

Diabetes is a major public health concern and is responsible for a huge social cost in both developed [1, 2] and low-income countries [3]. Disease management for diabetes (DMD) has long been thought to be effective in preventing disease-related complications and to subsequently lead to cost savings [4–7], although the results of existing studies have been mixed because of poor design of interventional programs, small sample sizes, and relatively short evaluation periods to detect changes in disease prognosis [8]. Recently, the systematic introduction of a disease management program in the public health insurance scheme in Germany has shown improvements in the process of care, whereas mixed results have been reported in terms of the impact on health outcomes [9, 10]. These descriptive observational studies in Germany lacked a comparable control group and were therefore vulnerable to selection bias. Furthermore, these studies did not report the cost-saving impact of the German DMD program.

Several econometric studies have been conducted to empirically evaluate the impact of DMD programs on health outcomes and cost savings. Dusheiko et al. [11] examined DMD based in primary care in the United Kingdom using panel data analysis to account for heterogeneity between the treated and control groups; they found DMD to have only a limited impact on hospital cost reduction. Kranker [12] employed the difference-in-differences (DD) technique with fixed-effects estimators, finding that DMD interventions for those at higher risk of disease-related complications reduced medical expenditures for diabetes treatment by 8.3% in the Georgia Medicaid program in the United States.

These previous studies have left the mechanism of cost savings unexplored. They have adopted a before/after-intervention two-period model to compare cost change between treatment and control groups. DMD interventions aim to correct the process of care, which may be under-used or over-used before the intervention. It is expected that correcting the process of care will improve disease prognosis by preventing disease-related complications such as macrovascular complications (e.g., stroke, heart attack, and chronic arterial obstruction), microvascular complications (e.g., nephropathy and retinopathy), and neuropathy. Thus, cost savings could be derived from 1) the rectification of the care process and 2) improved disease prognosis, which is endogenously determined by the corrected process of care. Previous mixed results for cost savings might be expected to occur, given that the pre-interventional process of care is sometimes over-utilized (likely in the case of the United States) and sometimes under-utilized (likely in the case of the United Kingdom). Indeed, Kranker found that cost savings were observed in the first 6–12 months after the introduction of DMD and that costs were then constant [12], suggesting that care process rectification, rather than health improvement, was responsible for the change.

In the present study, we sought to explain the mechanism of cost savings resulting from a DMD intervention, using panel data derived from the Japanese public health insurance scheme with fee-for-service payments, which is susceptible to overutilization. We focused on a DMD program organized by local public health insurers that has already been shown to be clinically effective for preventing diabetic nephropathy. We adopted a new three-period model to discriminate cost savings resulting from the rectification of the care process from cost savings caused by improved disease prognosis; we further describe the rationale of this model below.

### Kure National Health Insurance’s DMD program

Japan’s universal public health insurance system consists of over 3400 independent public insurers, including 1716 municipality-operated public insurers working under the National Health Insurance (NHI) policy protocol. The NHI covers self-employed and retired people, who account for 28.3% of the total population in Japan [13].

The city of Kure is located in Hiroshima Prefecture, in western Japan. Kure is the third largest city in the prefecture, with a population of 242,252 [14]. In 2010, the Kure NHI introduced a DMD program developed by Hiroshima University specifically to reduce treatment costs related to diabetes-induced nephropathy and subsequent hemodialysis. Diabetic nephropathy is the leading cause of hemodialysis treatment in Japan and is a major source of the costs caused by poor diabetes control: These costs represented an average medical expenditure per patient per month of 479,000 yen (about 5000 United States dollars [USD]) in 2010 [15].

Kazawa and Moriyama [16] have provided a detailed description of the Kure DMD program. Briefly, trained nurses conduct risk assessments of beneficiary patients diagnosed with diabetes and advise the patients on self-management and care through face-to-face interviews to coordinate appropriate medical services in collaboration with family physicians. The same nurses conduct one-hour face-to-face education sessions in the patients’ homes or at community centers, at the patients’ convenience, every 2 weeks for 2 months after enrollment, followed by monthly telenursing education until the 6th month from the beginning of the intervention. In this study, we defined the intervention period as 6 months. Data on body weight, blood pressure, physical conditions, diet, and exercise, as well as other laboratory parameters, were collected and shared among the program team. The patients’ physicians received monthly progress reports from the nurses, as well as immediate alert messages if any abnormal data or symptoms were detected.

Kazawa and Moriyama [16] and Kazawa et al. [17, 18] have reported that this DMD program in the Kure NHI has led to significant improvements in biomarkers (e.g., HbA1c and estimated renal function) 6 months after the intervention period, as well as to reductions in the risk of chronic renal failure requiring hemodialysis treatment.

The rest of this article is structured as follows. The data and empirical strategy for effect estimation are discussed in the second section. The estimation results are presented in the third section, and the implications of the results, as well as future challenges, are discussed in the fourth section. The conclusions are presented in the final section of the article.

## Methods

### Study subjects and data

We obtained monthly medical claims data provided by the Kure NHI for the 2010–2013 fiscal years. These claims data include information on types of medical services utilized, treatment costs based on standardized reimbursement prices, and patient information such as sex, age, postal code, and comorbid diagnoses.

We also obtained data on the DMD program participants (treatment group) and nonparticipants (control group) for the 2010–2013 fiscal years. Participants in the DMD program were selected in three steps. First, the public insurer (Kure NHI) selected candidates with stage 3 or higher diabetic nephropathy by checking prescription information from the medical claims data and confirming this diagnosis with the patients’ primary care physicians. Second, Kure NHI sought written consent from the candidates to participate in the DMD program, as well as permission/agreement from the candidates’ primary care physicians. Third, candidates became participants when both they and their physicians agreed to their participation in the program. These participants formed the treatment group in the present study. If either the candidate or his/her physician rejected the candidate’s participation in the program, that candidate was considered a nonparticipant and was assigned to the control group. For this study, we obtained data on 172 diabetic nephropathy patients in the treatment group and 2684 patients in the control group.

Finally, we combined the medical claims data and the DMD program intervention information using patients’ unique identification numbers, which were provided by Kure NHI. Patients who dropped out of the DMD program for any reason were treated on an intention-to-treat basis and were included in the analysis.

### Independent and dependent variables

We used two dependent variables. The most important of these was dialysis costs, which is the main target of the DMD program. We were also interested in the additional cost-containment effect with regard to related diseases. Therefore, we also examined the total medical claims costs excluding the costs of dialysis as a second dependent variable. These two dependent variables were transformed into their logarithmic forms.

Patients with diabetes tend to have several diabetic complications and other non-diabetic comorbidities that strongly influence their prognosis over time as a time-varying covariate. We adopted the Charlson comorbidity index to classify patients’ prognostic morbidity by generating a weighted index that takes into account the number and seriousness of comorbid diseases, following Quan’s protocol [19, 20].

There are three regions in the city of Kure: Hondo, Ondo, and Kamagari. Using the patient’s postal code, we created two dummy variables for region (for the regions of Ondo and Kamagari) to control for patient characteristics related to residential area as a time-invariant covariate.

We also generated two income earner dummy variables to control for patient characteristics based on their income earner status in the household (primary or secondary). For example, in the case of one typical type of household, the full-time worker father would be the primary income earner, and the part-time worker mother would be the secondary earner. Other household members (children and the retired older adult parents of the income earners) would be in the reference group for comparing with both primary and secondary earners. Unfortunately, we were unable to gain permission from Kure NHI to use the patients’ household income information in this study.

Descriptive statistics for the model variables are shown in Table 1.

**Table 1:** Descriptive statistics

Variable Description	Mean	Std. Dev.	Min	Max	Obsevations	Individuals
Dialysis cost (Yen)	63,760	454,159	0	8,848,000	80,297	2856
Total medical claim cost (Yen)	86,745	205,162	70	6,885,340	80,297	2856
T:treatment group dummy variable (β1)	0.012	0.110	0	1	80,297	2856
A:after intervention dummy variable (β2)	0.163	0.369	0	1	80,297	2856
TA: treatment group and after intervention dummy variable (β3)	0.007	0.086	0	1	80,297	2856
TB: treatment group and before intervention dummy variable (β4)	0.010	0.101	0	1	80,297	2856
B: before intervention dummy variable (β5)	0.137	0.344	0	1	80,297	2856
Female gender dummy variable	0.544	0.498	0	1	80,297	2856
Age	66.336	6.365	19	75	80,297	2856
Charlson comorbidity index scores	2.242	2.052	0	28	80,297	2856
Ondo dummy variable	0.100	0.300	0	1	80,297	2856
Kamagari dummy variable	0.052	0.221	0	1	80,297	2856
primary income earner dummy variable	0.349	0.477	0	1	80,297	2856
secondary income earner dummy variable	0.093	0.290	0	1	80,297	2856

Note: All monetary values are in Japanese yen

### Empirical approach

Our empirical strategy was to use estimators of the effects of the DMD program to account for time-varying and time-invariant heterogeneity at the individual level between the treatment and control groups. We controlled for time-invariant heterogeneity by using fixed effects panel analysis, following Dusheiko et al. [11] and Kranker [12]. To study the robustness of the estimation method, we also used the random-effect model and the fixed-effect model with and without robust standard errors.

**Fig. 1:**
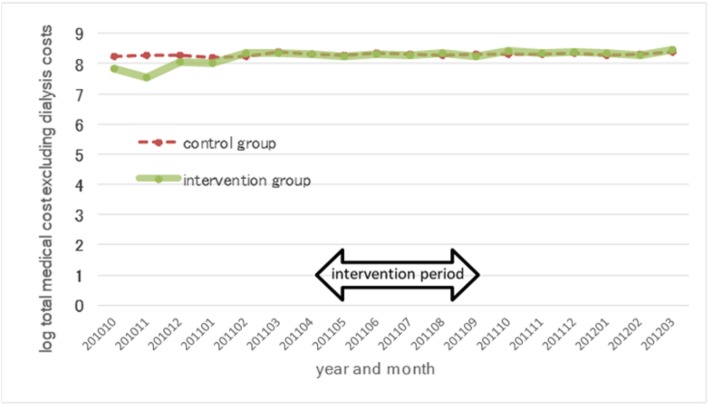
Average log total medical cost excluding dialysis cost per patient per month of DMD participants and non-participants in the 2011 fiscal year. The vertical axis represents the log-transformed average value of the total medical cost excluding dialysis cost, and the period (month and year) is shown on the horizontal axis

**Fig. 2:**
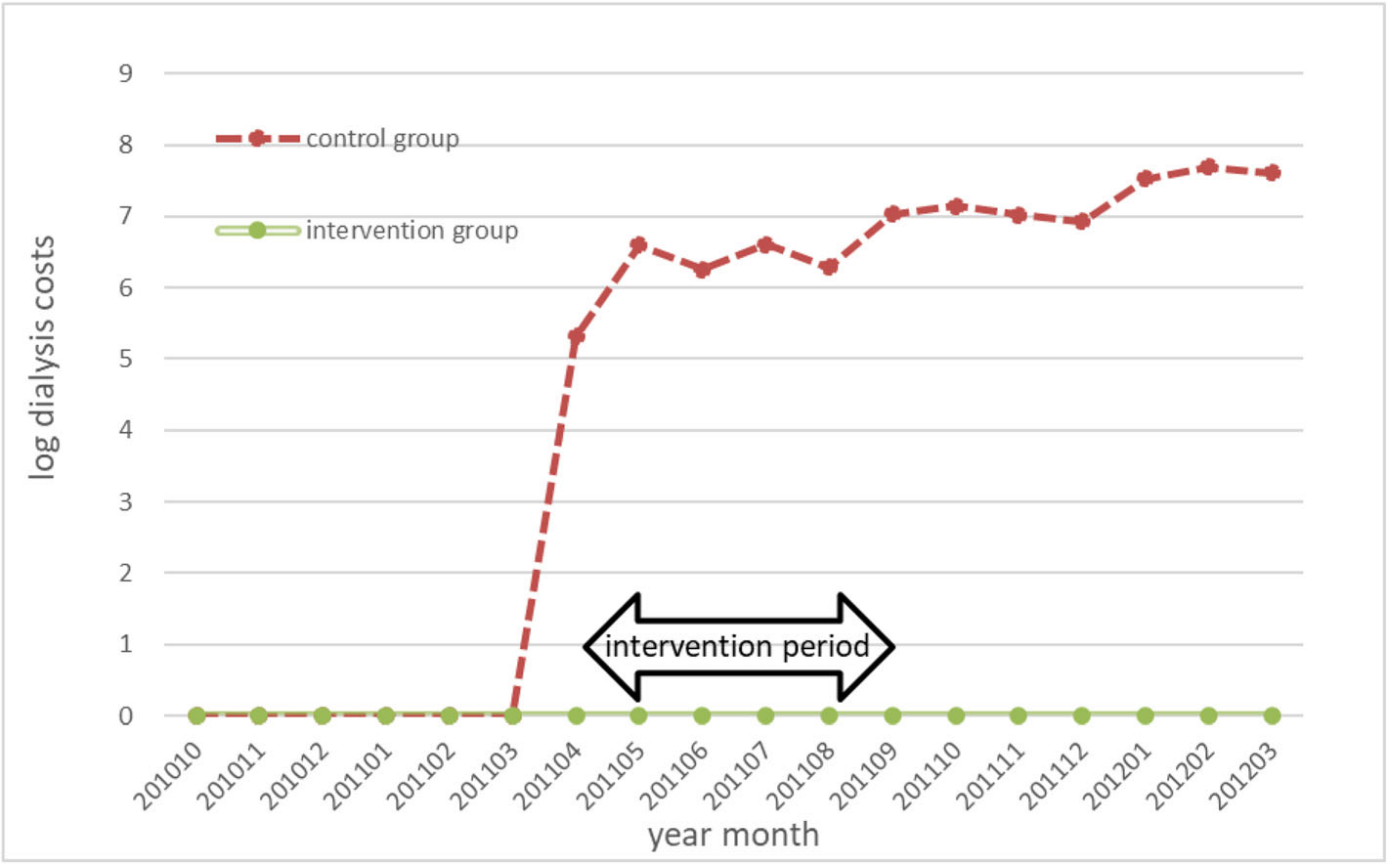
Average log dialysis cost per patient per month of DMD participants and non-participants in the 2011 fiscal year. The vertical axis represents the average log-transformed value of the dialysis cost, and the period (month and year) is shown on the horizontal axis. Both the intervention group and the control group had a value of zero before the intervention period

We adopted the DD estimation strategy to evaluate the program impact on cost savings. This approach allowed us to examine the influence of the program intervention, eliminating external influences, such as changes in the fee schedules for reimbursement by public health insurance. We needed to satisfy the “parallel trends” assumption to obtain unbiased DD estimators [21]. Our examination of the changes in mean monthly expenditures per patient in the pre-intervention period suggested parallel pre-intervention trends in both groups (Figs. 1 and 2).

Fig. 1 comprises trend lines of the mean log total medical cost excluding the cost of dialysis per patient per month from October 2010 to March 2012 for both DMD program participants and DMD program non-participants in Kure. Figure 2 comprises trend lines of the mean log dialysis cost per patient per month from October 2010 to March 2012 for both DMD program participants and DMD program non-participants in Kure. The intervention period started in April 2011. For log total medical cost excluding dialysis costs, the pre-intervention trends in the treatment group and the control group would show parallel trends in the two groups. For log dialysis cost, both the intervention group and the control group had a value of zero before the intervention period.

**Fig. 3:**
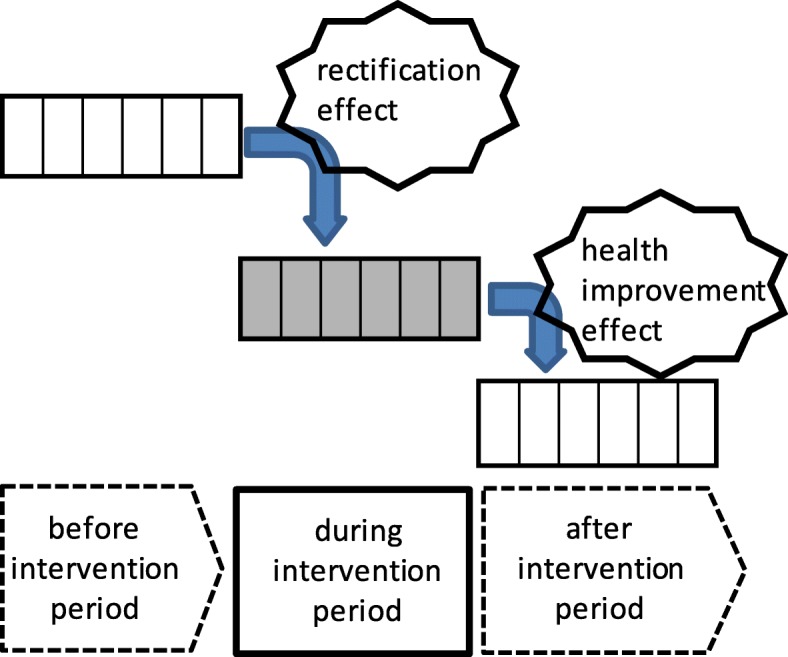
Hypothesized distinctive effects in the three-period difference-in-differences analysis. Each of the three periods in the three-period difference-in-differences analysis lasted 6 months

Finally, over- or under-medication sometimes occurs in health insurance systems with fee-for-service reimbursement where there is no gatekeeper. The problem of excess provision of medical services could be significant in Japan because of the fee-for-service-based reimbursement system [22]. Thus, we needed to consider two effects separately in the DMD program evaluation. The first effect—the “rectification effect”—refers to the medical cost reduction resulting from case management in the DMD program through the elimination of excess or redundant medical services. This effect was expected to be seen in the cost difference between the before-intervention period and the during-intervention period. The second effect—the health improvement effect—refers to the medical cost reduction resulting from the maintenance of the condition of diabetic nephropathy and the prevention of the aggravation of diabetic nephropathy by the DMD program. This effect was expected to be observed in the cost difference between the during-intervention period and the after-intervention period (Fig. 3). To distinguish between the cost-containment effects of health improvement and of modification of over- or under-medication, it was necessary to compare three periods: In addition to the before-intervention period and the after-intervention period, it was crucial to examine the *during*-intervention period. We therefore expanded the DD analysis from two to three periods, as is described in the next section. We set the duration of both the before-intervention period and the after-intervention period at 6 months to match the 6-month (during-) intervention period.

### Expansion of DD analysis from two to three periods

The standard model for the DD design is as follows. Y_it_ represents the outcome for individual *i* at time *t*. The treatment group dummy is represented by *treat*_*i*_, which is 1 if a person is in the treatment group and is 0 otherwise. The time dummy variable is represented by *time*_*t*_, which is 1 if the time is after treatment and is 0 otherwise. In the standard DD model, the outcome for individual *i* at time *t* satisfies Equation (1):

1 $$ {\mathrm{Y}}_{\mathrm{i}\mathrm{t}}={\upbeta}_0+{\upbeta}_1\left({\mathrm{t}\mathrm{reat}}_{\mathrm{i}}\right)+{\upbeta}_2\mathrm{A}\left({\mathrm{t}\mathrm{ime}}_{\mathrm{t}}\right)+{\upbeta}_3\left({\mathrm{t}\mathrm{reat}}_{\mathrm{i}}\cdotp {\mathrm{t}\mathrm{ime}}_{\mathrm{t}}\right)+{\varepsilon}_{\mathrm{i}\mathrm{t}} $$

The third coefficient, β_3_, represents a group-specific, time-specific effect. The last term, ε_it_, represents an individual’s unobservable characteristics. This term is assumed to be independent of the group indicator and to have the same distribution over time.

As introduced above, we propose an expansion of the standard model from two to three periods. The time dummy variable (*time*_*i*_) can take the value 0, 1, or 2. We categorized the time dummy variable into two types—the before- (B) and after-intervention (A) dummy variables. The before-intervention time dummy variable is 1 when a patient is in the before-intervention period and is 0 otherwise. The after-intervention time dummy variable is 1 when a person is in the after-intervention period and is 0 otherwise. The empirical model specification for the three-period DD analysis is shown in Equation (2):

2 $$ {\displaystyle \begin{array}{l}{\mathrm{Y}}_{\mathrm{i}\mathrm{t}}={\upalpha}_{\mathrm{i}}+{\upbeta}_1{\mathrm{t}\mathrm{reat}}_{\mathrm{i}}+{\upbeta}_2{\mathrm{after}\ \mathrm{intervention}}_{\mathrm{t}}+{\upbeta}_3\left({\mathrm{t}\mathrm{reat}}_{\mathrm{i}}\cdotp {\mathrm{after}\ \mathrm{intervention}}_{\mathrm{t}}\right)\\ {}\kern1.44em +{\upbeta}_4\left({\mathrm{t}\mathrm{reat}}_{\mathrm{i}}\cdotp {\mathrm{before}\ \mathrm{intervention}}_{\mathrm{t}}\right)+{\upbeta}_5{\mathrm{before}\ \mathrm{intervention}}_{\mathrm{t}}+{\Sigma \upbeta}_{\mathrm{k}}{X}_{\mathrm{i}\mathrm{t}}+{\varepsilon}_{\mathrm{i}\mathrm{t}}\end{array}} $$

where Y_it_ represents the outcome as the medical costs per patient per month in the form of a logarithmic value and α_i_ is the individual fixed-effect variable. There are five kinds of dummy variables. First, *treat*_*i*_ is the treatment group dummy variable, as in the standard DD estimation. Both the after- (A) and the before-intervention (B) dummy variables were described above. Fourth, the treatment group and after-intervention dummy variable (TA) is a cross-dummy variable that indicates both the treatment group and the after-intervention period. Fifth, the treatment group and before-intervention dummy variable (TB) is a cross-dummy variable indicating both the treatment group and the before-intervention period. We also added several control variables as Σβ_k_*X*_it_.

Table 2 shows that -β_4_ represents the difference between the control and treatment groups by comparing the before- and during-intervention periods (the rectification effect). β_3_ represents the difference between the control and treatment groups by comparing the during- and after-intervention periods (the health improvement effect). β_3−_β_4_ represents the difference between the two groups by comparing the before- and after-intervention periods (both effects). Therefore, we can distinguish the DMD program’s cost-containment effect caused by the rectification of overtreatment between the before-intervention period and the DMD-intervention period. The statistical analysis was performed using Stata/IC, version 11.0.

**Table 2:** Effects indicated by the regression coefficients

	Difference between before and during the intervention	Difference between during and after the intervention	Difference between before and after the intervention
Coefficient for the treatment group	-*β*4 − *β*5	+*β*2 + *β*3	+*β*2 + *β*3 − *β*4 − *β*5
Coefficient for the control group	-*β*5	+*β*2	+*β*2 − *β*5
Difference between the treatment and control groups	-*β*4 (the rectification effect)	+*β*3 (the health improvement effect)	-*β*4 + *β*3 (both effects on costs)

## Results

### Cost-containment effect on dialysis cost

We estimated the cost functions using the random-effect model, the fixed-effect model, and the fixed-effect model with robust standard errors. We estimated regression models to predict two dependent variables, dialysis costs and total medical claims costs, to examine the effects (Table 3).

**Table 3:** Estimation results of the three-period difference-in-differences analysis

Dependent Variable		Dialysis cost		Total medical claims costs (excluding dialysis costs)
Estimation method	Random Effect	Fixed Effect	Fixed Effect (robust)	Fixed Effect (robust)
	Estamated coefficient [z-statistics]	Estimated coefficient [t -statistics]	Estimated coefficient [t -statistics]	Estimated coefficient [t -statistics]
T: treatment group dummy variable (β1)	−0.0098 [− 0.50]	− 0.0086 [− 0.44]	−0.0080 [−1.77]	0.0432 [1.31]
A: after-intervention dummy variable (β2)	0.0160 [3.00]**	0.0161 [3.02]**	0.0160 [1.63]	0.0383 [3.87]**
TA: treatment group and after-intervention dummy variable (β3)	−0.0310 [−1.25]	− 0.0300 [− 1.21]	−0.0299 [−2.13]*	−0.0886 {−2.49]*
TB: treatment group and before-intervention dummy variable (β4)	0.0397 [1.82]	0.0411 [1.88]	0.0410 [2.72]**	−0.0158 [− 0.47]
B: before-intervention dummy variable (β5)	−0.0474 [−8.02]**	−0.0475 [− 8.04]**	−0.0475 [−3.31]**	−0.0477 [− 4.63]**
Female sex dummy variable	− 0.0274 [− 0.36]	Omitted	Omitted	Omitted
Age	−0.0449 [− 9.56]**	Omitted	Omitted	Omitted
Charlson comorbidity index score	0.0049 [3.66]**	0.0043 [3.24]**	0.0040 [1.16]	0.0812 [20.37]**
Ondo dummy variable	−0.0894 [− 0.089]	Omitted	Omitted	Omitted
Kamagari dummy variable	0.1206 [0.91]	Omitted	Omitted	Omitted
Primary income earner dummy variable	−0.0584 [− 0.75]	Omitted	Omitted	Omitted
Secondary income earner dummy variable	0.6652 [6.30]**	Omitted	Omitted	Omitted
Constant	3.1846 [9.75]**	0.2479 [65.88]**	0.2479 [28.49]**	8.1396 [882.56]**
Diagnostic Test				
Number of observations	80,297	80,297	80,297	80,297
Number of individuals	2856	2856	2856	2856
R2 within	0.0012	0.0012	0.0012	0.0253
between	0.0387	0.0044	0.0044	0.0851
overall	0.0424	0.0014	0.0014	0.0512
sigma_u	1.6007	1.6457	1.6457	0.7619
sigma_e	0.5236	0.5235	0.5235	0.7176
rho	0.9033	0.9080	0.9080	0.5298

Notes: All estimation models are panel data estimation models. “Omitted” means that Stata statistical software automatically omitted several independent variables. * indicates significance at the 5% level, and ** indicates significance at the 1% level. The“sigma_u” is the standard deviations of residuals within groups, and the “sigma_e” is the standard deviation of residuals overall. The rho is the interclass correlation of the above two values. The rho value (0.908) tells us that 90.8% of the variance is because of difference across time (within units)

In the two fixed-effect estimation models, all time-invariant explanatory variables (e.g., sex, age, the region dummy variable, and the income earner dummy variable) were omitted. As a control variable, the Charlson comorbidity index was statistically significant and met our expectations in both the random-effect model and the fixed-effect model without robust standard errors, but not in the fixed-effect model with robust standard errors.

We conducted several statistical tests to select the estimation method, including the Breusch and Pagan Lagrangian multiplier test for random effect, the *F*-test, the Hausman test, and the modified Wald test for groupwise heteroskedasticity in fixed-effect regression models [23]. Based on the results, we selected the fixed-effect model and adopted robust standard errors (cluster-robust at patient level).

The DMD program’s main purpose is to prevent the introduction of dialysis; therefore, we first checked the results for dialysis costs (Table 3). In the model predicting the dependent variable of dialysis costs, the coefficient of the treatment group and before-intervention (TB) dummy variable (β_4_) was 0.0410 and was statistically significant. This result indicates that the rectification effect on dialysis costs was − 0.0410. The coefficient of the treatment group and after-intervention (TA) dummy variable (β_3_) was − 0.0299 and was also statistically significant. This result indicates that the health improvement effect on dialysis costs was − 0.0299. In sum, we find that the overall magnitude of the DMD program effects on these costs was − 0.0709 (β_3_ − β_4_).

Because we included the dependent variables in the estimation models as logarithmic transformations, the numerical values of the coefficients had to be logarithmically inverse transformed to evaluate the intervention’s impact on costs. The rectification effect accounted for a 4.02% reduction in dialysis costs from the before-intervention period to the during-intervention period, and the health improvement effect accounted for a 2.95% reduction in dialysis costs from the during-intervention period to the after-intervention period. In sum, we found that the total magnitude of the DMD program’s effect on medical expenditures was − 6.84%.

### Cost-containment effect on all medical claims costs (excluding dialysis costs)

We also estimated a model predicting the dependent variable of total medical claims costs excluding dialysis costs to evaluate additional cost-containment effects of the DMD program. The results showed that the coefficient of the treatment group and before-intervention (TB) dummy variable (β_4_) was insignificant, whereas the coefficient of the treatment group and after-intervention (TA) dummy variable (β_3_) was − 0.0889 and statistically significant. Therefore, the rectification effect on non-dialysis medical claims costs was nil, and the health improvement effect on these costs was − 0.0889; these results indicate that the magnitude of the DMD program’s effect on the total non-dialysis medical claims costs was − 0.0889 (β_3−_β_4_).

After antilogarithm calculation, the health improvement effect accounted for an 8.51% reduction in total non-dialysis medical claims costs from the during-intervention period to the after-intervention period. However, we could not determine any rectification effect on total non-dialysis medical claims costs. These results showed that the total magnitude of the DMD program’s effect on the total non-dialysis medical claims costs was − 8.51%. Therefore, we found evidence of an additional cost-containment effect of the DMD program not only for dialysis costs but also for other medical expenditures.

## Discussion

### Significant reduction in dialysis costs

This study found that the DMD program had negative and significant effects on medical expenditures. The rectification effect accounted for a 4.02% reduction in dialysis costs, and the health improvement effect accounted for a 2.95% reduction in dialysis costs. We tested the equality of two coefficients representing the rectification effect and the health improvement effect and did not find a statistically significant difference between these two coefficients. Therefore, the magnitude of the two effects is not statistically different. An expected result was our finding of similar rectification (4.02%) and health improvement (2.95%) effects on dialysis costs. This result was likely caused by the characteristics of patients in our sample, who had relatively high-risk diabetes; practices characterized by a tendency toward overmedication under a fee-for-service reimbursement system; and/or less standardized treatment processes for patients with severe diabetes.

In this study, the DMD program was found to reduce dialysis costs by a total of 6.84% for total medical expenditures per patient per month. However, a review of the economic impact of disease management found that only half of the studies on this topic reported cost savings [24].

In the United Kingdom, Dusheiko et al. [11] found no statistically significant impact of diabetes care on hospital costs. They examined whether higher quality disease management in primary care was associated with reduced hospital costs, using fixed-effect and random-effect models. Specifically, they examined the relationships between Quality and Outcomes Framework scores, which are proxy variables for the quality of disease management, and hospital costs in England. They found a statistically significant negative association between the Quality and Outcomes Framework stroke quality score and patients’ hospital costs. However, in the case of diabetes, the relationship was unclear. The need for inpatient care, such as emergency hospitalization, would be smaller for patients with diabetes than it would be for patients with stroke, all else being equal. Therefore, it would be very difficult to detect a cost-reduction effect on secondary care from the quality of DMD in the primary care sector.

In the United States, Kranker [12] found that DMD for those at higher risk of disease-related complications reduced medical expenditure for diabetes treatment in the Georgia Medicaid program, showing a cost-containment effect of 8.3%, which was slightly higher than the effect found in the present study. This difference may be attributed to a difference in patients’ severity of disease between our study and this previous study. Our sample was limited to very severe cases with stage 3 or higher diabetic nephropathy. Another factor that may explain this difference is related to the difference of the reimbursement systems in Japan and the United States. The lower prices of medical services reimbursed through the public health insurance in Japan result in lower effect sizes in terms of the reduction in medical costs.

However, the direct cost of the DMD intervention was 270,000 yen per person (about USD 2077 in terms of purchasing power parity). From this estimated result, we expect both dialysis cost reduction and other medical cost reduction to be a total of 70,404 yen per person per 6-month period (about USD 542). If the cost-saving effect persists for more than 2 years (four 6-month periods) after the intervention period, this medical cost containment (USD 2168) could surpass the direct cost of the DMD intervention (USD 2077).

### Study limitations and further research questions

This study had several limitations that should be resolved in future work. First, the sampling process may have resulted in selection bias, especially in the intervention group. In the selection of participants for the DMD program, both the patients and their primary care physicians had to give their approval or agreement. Primary care physicians may make different decisions because of differences in practice styles; thus, they may come to different conclusions about whether to approve their patients’ participation in the DMD program. For example, some doctors consider prevention less important than treatment and thus may be more likely to disapprove of their patients’ participation in the DMD program. This situation may have introduced some bias in the sample allocation of participants to the DMD program (the intervention group). However, if the differences in physicians’ practice styles are randomly distributed in the treatment and control groups, the magnitude of the bias would be insignificant. Differences in the practice styles of primary care physicians also affect unobserved patient characteristics that may have influenced the probability of enrollment in the DMD program. This potential selection bias is not randomly distributed in the treatment and control groups; however, under the parallel trends assumption, the DD estimates allow us to identify the average treatment effect on the treated.

The second limitation involves the potential selection bias that remained in the two groups. The absence of patient blinding (a placebo) in our study may have resulted in some bias in our estimation of the treatment effects. Wood et al. [25] claimed that the estimation of intervention effects is exaggerated in non-blinded trials, compared with blinded trials. However, their study also found that the bias associated with the absence of patient blinding seemed to be restricted to trials with subjectively assessed outcomes.

The third limitation is the potential underestimation of the effects on costs because of our short-term observation period (each of the three periods was 6 months). Dialysis costs are a long-term burden for public insurers in Japan, so long-term effects on costs should be investigated. However, a DD analysis is suitable for evaluating short-term effects. Further studies should prolong the observation period and increase the sample size to generalize our study’s results.

The fourth limitation of the study is the underestimation of dialysis costs because of data censoring, which occurred when some people who were insured by the Kure NHI moved to other public health insurers after reaching a certain age. All Japanese people aged 75 years and over and those aged 65 to 74 years who have disabilities mandatorily move to public health insurance exclusively for older adults (the Late-Stage Medical Care System for the Elderly). We were unable to track participants or nonparticipants after they moved away from Kure NHI to this system. Our sample’s average censoring rate was 19.9% during the study period. Further studies should consider expanding these censored data in an appropriate manner.

The fifth limitation is that our models explaining a part of the medical costs had relatively low coefficients of determination. This limitation could be caused by missing variables, such as income, which we were unable to use in our estimation models. Thus, care should be taken in generalizing the study’s results.

## Conclusion

This study evaluated the cost-reduction effects of a DMD program for diabetic nephrology that aimed to prevent the necessity of dialysis. The cost-reduction effect in terms of treatment costs from the before-intervention period to the during-intervention period (the rectification effect) was 4.02%, and the cost-saving effect from the during-intervention period to the after-intervention period (the health improvement effect) was 2.95%. The examined disease management program for diabetes patients organized by local public insurers in Japan reduced costs, both by amending treatment processes and by subsequently improving the prognosis of the disease.

## Data Availability

All data in this study received permission from National Health Insurance, Kure City, as part of health-care measures for the insured. Furthermore, the dataset of this study cannot be shared due to confidentiality..

## Abbreviations

DMD: A disease management program for diabetes patients
NHI: National Health Insurance
DD: Difference-in-differences analysis
USD: United States dollars
